# Relationship between body composition and bone mineral density in postmenopausal women with type 2 diabetes mellitus

**DOI:** 10.1186/s12891-022-05814-8

**Published:** 2022-10-03

**Authors:** Lei Gao, Ping Zhang, Yan Wang, Wei Zhang, Jian Zhao, Ying Liu, Jing Liu, Shaoqiang He

**Affiliations:** 1 Department of CT/MRI, the Third Hospital of Hebei Medical University, No. 139 Ziqiang Road, Qiaoxi District, Shijiazhuang, 050051 Hebei Province China; 2 Department of Endocrinology, the Third Hospital of Hebei Medical University, No. 139 Ziqiang Road, Qiaoxi District, Shijiazhuang, 050051 Hebei Province China; 3 Department of Radiology, the Third Hospital of Hebei Medical University, No. 139 Ziqiang Road, Qiaoxi District, Shijiazhuang, 050051 Hebei Province China

**Keywords:** Adipose tissue, Bone mineral density, Body composition, Type 2 diabetes mellitus

## Abstract

**Background:**

The aim of the study were to analyze the lumbar volumetric bone mineral density (BMD), fat distribution and changes of skeletal muscle with quantitative computed tomography (QCT) in postmenopausal women with type 2 diabetes mellitus (T2DM), and to evaluate the relationship between body composition and BMD.

**Methods:**

One hundred seventy-seven postmenopausal women with T2DM and 136 postmenopausal women without diabetes were included in the study and were divided into two groups according to age, 50–65 years age group and over 65 years of age group. The lumbar BMD (L1-L3), visceral fat mass (VFM), visceral fat area (VFA), subcutaneous fat mass (SFM), subcutaneous fat area (SFA), psoas major mass (PMM) and psoas major area (PMA) of each group were compared. Univariable and multivariable linear regression analysis were used to analyze the contribution of each variable to BMD in postmenopausal women with T2DM.

**Results:**

In women aged 50–65, the patients in the T2DM group had higher body mass index (BMI), VFM, VFA, and SFM (*p* < 0.05), compared with non-T2DM group. Over 65 years old, the BMI, BMD, VFM, VFA, and SFM was found to be much higher in participants with T2DM than in non-T2DM group (*p* < 0.05). Compared with women aged in 50–65 years old, those over 65 years old had higher VFA and VFM and lower BMD (*p* < 0.05), whether in the T2DM group or the non-T2DM group. Age, VFA and VFM were negatively correlated with BMD (*r* = -0.590, *p* ≤ 0.001; *r* = -0.179, *p* = 0.017; r = -0.155, *p* = 0.040, respectively). After adjusting for age, VFM and VFA were no longer correlated with BMD. No correlations between fat distribution or psoas major muscle and BMD in postmenopausal women with T2DM were observed.

**Conclusions:**

T2DM can affect abdominal fat deposition in postmenopausal women. Postmenopausal elderly women with diabetes have higher BMD than normal elderly women. There was no correlation between fat distribution or psoas major and BMD in postmenopausal women with diabetes mellitus.

**Supplementary Information:**

The online version contains supplementary material available at 10.1186/s12891-022-05814-8.

## Background

In 2017, there were approximately 451 million people suffer from diabetes globally, and this number is projected to increase to 693 million by 2045, of which more than 90% of the patients were type 2 diabetes mellitus (T2DM) [1]. China has the largest number of patients with T2DM in the world, which continues to increase [2]. Studies have shown that abdominal obesity, sarcopenia and bone mineral density (BMD) are associated with insulin resistance (IR), which is the characteristic of T2DM [3–5]. Although the BMD is normal or higher, the recognized fracture risk of T2DM complications has increased by 40%-70% [6], and potential factors have not been clearly identified, increased risk of falls, obesity, muscle loss and anti-diabetic drugs may be responsible for increased fracture risk [7, 8]. Aging usually leads to loss of lean mass (LM), increase of adipose tissue, and decrease of BMD, but there are few studies on these changes in diabetic patients. In addition, the relationship between body components such as muscle or fat and BMD is less studied in diabetic population.

Previous studies have used dual-energy X-ray absorptiometry (DXA) to assess BMD and body composition [9]. Although DXA is considered to be the gold standard for the diagnosis of osteoporosis, quantitative computed tomography (QCT) which measures the volume of BMD which is the true trabecular BMD can avoid the influence of vertebral osteophyte, facet degeneration, intervertebral disc stenosis, endplate sclerosis and abdominal aortic wall calcification, so as to evaluate the vertebral BMD more truly and accurately, compared with DXA [10, 11]. As a three-dimensional measurement method for BMD, QCT improves the sensitivity and accuracy of BMD measurement, compared with DXA [11, 12]. At the same time, QCT can obtain the corresponding fat and muscle content. Few studies have evaluated body compositions such as abdominal adipose, visceral fat content and changes of skeletal muscle in T2DM patients using QCT. Therefore, in the present study, we aimed to investigate the effects of T2DM on BMD, visceral and subcutaneous fat, and psoas major by QCT in postmenopausal women over 50 years of age with T2DM.

## Methods

### Patients

This is a retrospective study involving 385 postmenopausal females admitted in department of endocrinology of our hospital. All participants underwent QCT examination because of low back pain or numbness and pain of lower limbs in our hospital. The inclusion criteria included T2DM postmenopausal females and postmenopausal females without diabetes. Postmenopausal was defined as at least one year without a menstrual period. T2DM was defined according to the updated and approved diagnostic criteria for diabetes of the ADA released in 2022 [13], patients with fasting plasma glucose (FPG) ≥ 126 mg/dL (7.0 mmol/L) (no caloric intake for at least 8 h) or 2-h posted-glucose (PG) ≥ 200 mg/dL (11.1 mmol/L) using oral glucose tolerance test (OGTT) or glycosylated hemoglobin (HbA1c) ≥ 6.5% (48 mmol/mol) or typical symptoms of hyperglycemia or hyperglycemic crisis, random blood glucose ≥ 200 mg/dL (11.1 mmol/L). Postmenopausal female participants who met the diagnostic criteria for T2DM were included in the T2DM group. Others who did not meet the diagnostic criteria of T2DM were included in the non-T2DM group. The exclusion criteria included history of hysterectomy, malignant tumor, vertebral compression fracture, smoking and drinking history, taking drugs affecting bone metabolism such as sex steroids, warfarin and bisphosphonates, suffering from diseases that influence bone metabolism including renal failure, hyperthyroidism and hyperparathyroidism, and history of paralysis due to other diseases. In addition, all participants did not take oral thiazolidinediones that may lead to bone loss in the treatment of diabetes [14]. All patients were categorized into two groups according to their ages (50–65 and > 65 years old) in the T2DM group and the non-T2DM group, respectively, to explore how the BMD and body compositions change and their relationship. Height and weight were documented to calculate the BMI (kg/m^2^). The treatment, levels of HbA1c and diabetic duration of T2DM participants were recorded.

Ethics committee of our institution approved the study.

### QCT parameter measurement

All participants underwent CT scan of lumbar vertebrae with a 64-slice CT scanner (Siemens 64 spiral CT, Germany) with hands raised above the head and with a solid-state QCT calibration phantom (Mindways Software Inc., Austin, TX, USA) closely beneath every patient low back simultaneously. Scan parameters were tube voltages,120 kV; tube current, 125 mAs; pixel, 0.78mm^2^; pitch, 0.8 mm; table height, 168 cm; matrix, 512 × 512; scanning field of view (SFOV), 500 mm; and thickness, 1 mm. The phantom was placed at the level of thoracic 12 to the 5th lumbar vertebra. Reconstruction parameters were standard algorithm, 1-mm section thickness and interval, and 400 mm display field of view. The scanning range was from the upper edge of 12 thoracic vertebrae to the lower edge of the 5th lumbar vertebra in the supine position.

Images were transferred to a QCT workstation and analyzed using the three-dimensional (3D) spine function version 5.10 of Mindways QCT pro soft-ware (Mindways Software Inc., Austin, TX, USA). Elliptical regions of interest (ROI) was placed at the midplane of lumbar 1–3 vertebral body to avoid the influence of cortical bone and proliferative osteophyte. The area of ROI is about 250mm^2^. Each parameter was calculated individually by two radiologists. Area and mass of abdominal visceral fat and subcutaneous fat and psoas major muscle of the middle level of L3 vertebral body was measured.

### Body compositions measurement

Measurements of visceral fat mass (VFM), visceral fat area (VFA), subcutaneous fat mass (SFM), subcutaneous fat area (SFA), psoas major mass (PMM) and psoas major area (PMA) were semi-automatically and manual adjustment completed by the commercial software package with a reconstruction thickness of 1 mm.

### Statistical analysis

The SPSS 26.0 statistical software package (IBM Corp., Armonk, NY, USA) was used for statistical analysis. Statistical description of the quantitative variables throughout the study were expressed as mean ± standard deviation (SD) for normal distribution data or medians (interquartile range; IQR) for non-normal distribution data. The Shapiro–Wilk test was used to test whether the data accord with the normal distribution. One-way ANOVA or Kruskal Wallis non-parametric test was used to test the differences of variables between different groups. The correlations between BMD and body composition variables were analyzed with the Pearson correlation test for normally distributed variables and Spearman correlation test for non-normally distributed data. Univariable and multivariable linear regression model were developed to identify the factors contributing to BMD. In linear regression, the average BMD of lumbar 1–3 vertebrae was used as the dependent variable, while BMI, age and body composition were the independent variables. Collinearity diagnosis was performed before multiple regression analysis. Statistical significance was accepted when *p* < 0.05.

## Results

### Baseline characteristics of participants

Of the 385 participants included, 72 were excluded from the study because of history of taking anti-osteoporosis drugs (*n* = 20), fracture history of lumbar or hip (*n* = 28) and hysterectomy history (*n* = 24). Finally, there were 313 participants were enrolled in this study, including 177 postmenopausal females with T2DM aged 50–88 years (mean age 65.77 ± 9.75 year-old) and 136 postmenopausal females without diabetes aged 50-91 years (mean age 66.21 ± 9.64 year-old). The characteristics including HbA1c value and treatment of the whole participants are presented in Table 1.

**Table 1:** Baseline characteristics of study participants

	T2DM group	non-T2DM group	*P*-value
No. of participants	177	136	
Age (years)	65.96 ± 9.69	66.21 ± 9.64	> 0.05
HbA1C (%)	8.00 (7.00, 9.30)	None	NA
Diabetic duration (years)	11.50 ± 5.04	None	NA
Treatment			
Insulin	42 (23.73%)	None	NA
Insulin and Oral anti-diabetic agents, n (%)	55 (31.07%)	None	NA
Oral anti-diabetic agents (no thiazolidinediones), n (%)	80 (45.20%)	None	NA

Data were presented as number (percentage) for categorical data, (mean ± standard deviation) for parametrically distributed data or median (interquartile range) for nonparametrically distributed data

### Comparison of age, BMI, BMD, and body component between T2DM group and non-T2DM group

Participants with T2DM were classified into two groups based on the age in our study, of which 88 (28.1%) were at 50–65 years age and 89 (28.4%) were > 65 years age. In the non-T2DM group, there were 75 (24.0%) between 50–65 years old and 61 (19.5%) over 65 years old. Between the ages of 50 and 65, compared with non-T2DM group, the patients in the T2DM group had higher BMI, VFM, VFA, and SFM (*p* < 0.05). Other parameters, including age, BMD, SFA, PMM, and PMA were found to have no differences between the two groups. Over 65 years old, the mean ages of the patients in each group were similar. The BMI, BMD, VFM, VFA and SFM was found to be much higher in participants with T2DM than in non-T2DM group (*p* < 0.05), respectively. Although SFA in the T2DM group was relatively higher than that in the non-T2DM group, there was no statistical difference (Table 2).

**Table 2:** Comparison of clinical and QCT parameters between different groups

variables	T2DM		non-T2DM		*P*	*P*_*1*_	*P*_*2*_	*P*_*3*_	*P*_*4*_
	50-65y (*n* = 88)	> 65y (*n* = 89)	50-65y (*n* = 75)	> 65y (*n* = 61)					
Age (years)	59.00 (53.00, 61.00)	73.00 (69.50, 78.00)	60.00 (56.00, 62.00)	76.00 (70.00, 79.50)	< 0.001	< 0.001	< 0.001	0.366	0.550
BMI (kg/m^2^)	24.25 (21.65, 27.37)	24.90 (23.20, 27.95)	22.80 (21.10, 25.40)	23.80 (21.00, 25.45)	< 0.001	0.086	0.442	0.020	0.003
m-BMD (mg/cm^3^)	92.30 (66.50, 117.18)	55.00 (41.15, 74.55)	79.20 (57.50, 103.50)	37.50 (22.30, 59.90)	< 0.001	< 0.001	< 0.001	0.064	0.002
BMD-L1 (mg/cm^3^)	98.65 (73.98, 126.43)	61.10 (44.50, 82.50)	88.30 (59.00, 105.70)	44.90 (27.60, 63.90)	< 0.001	< 0.001	< 0.001	0.050	0.003
BMD-L2 (mg/cm^3^)	90.90 (65.45, 116.13)	54.80 (36.60, 72.50)	83.70 (54.20, 111.80)	37.00 (12.50, 56.65)	< 0.001	< 0.001	< 0.001	0.090	0.001
BMD-L3 (mg/cm^3^)	88.70 (63.43, 111,23)	54.40 (38.30, 69.25)	76.00 (58.00, 97.00)	36.80 (16.80, 58.75)	< 0.001	< 0.001	< 0.001	0.100	0.005
VFM (g)	35.25 (24.95, 44.98)	42.70 (33.90, 62.45)	25.30 (18.00, 32.40)	31.50 (19.45, 50.55)	< 0.001	0.001	0.003	< 0.001	< 0.001
VFA (cm^2^)	132.70 (100.15, 184.20)	160.90 (133.80, 229.45)	102.70 (77.20, 148.60)	134.50 (102.00, 194.45)	< 0.001	0.001	0.001	0.001	0.003
SFM (g)	38.90 (27.95, 54.58)	40.60 (28.35, 55.45)	27.10 (19.60, 41.30)	33.10 (14.95, 49.70)	0.001	0.861	0.323	0.001	0.031
SFA (cm^2^)	145.10 (110.68, 201.70)	151.50 (106.55, 206.00)	121.10 (92.20, 181.50)	145.10 (93.40, 199.85)	0.198	NA	NA	NA	NA
PMM (g)	5.60 (4.80, 7.15)	5.70 (4.35, 7.30)	5.60 (4.30, 6.80)	5.30 (4.25, 7.15)	0.503	NA	NA	NA	NA
PMA (cm^2^)	18.95 (16.20, 23.05)	18.50 (15.75, 23.90)	19.60 (16.90, 22.90)	19.30 (14.85, 24.00)	0.845	NA	NA	NA	NA

Values are presented as median (interquartile range). Kruskal–Wallis test was used for analysis

*T2DM* Type 2 diabetes mellitus, *BMI* Body mass index, *m-BMD* Mean bone mineral density, *BMD-L1* Bone mineral density of lumbar 1 vertebral body, *BMD-L2* Bone mineral density of lumbar 2 vertebral body, *BMD-L3* Bone mineral density of lumbar 3 vertebral body, *VFM* Visceral fat mass, *VFA* Visceral fat area, *SFM* Subcutaneous fat mass, *SFA* Subcutaneous fat area, *PMM* Psoas major mass, *PMA* Psoas major area

*P*_1_: 50-65y vs > 65y in T2DM group

*P*_2_: 50-65y vs > 65y in non-T2DM group

*P*_*3*_: 50-65y in T2DM group vs 50-65y in non-T2DM group

*P*_*4*_: > 65y in T2DM group vs > 65y in non-T2DM group

The BMD decreased with age and was significantly lower in participants aged > 65 years compared with those aged between 50 to 65 years, whether in the T2DM group or the non-T2DM group. VFM and VFA were higher at the age over 65 than at the age of 50–65 (*p* < 0.05), while there were no differences in terms of SFM, SFA, PMM and PMA (*p* > 0.05) (Table 2).

### Correlation between age, BMI, body component variables and BMD in T2DM group

**Fig. 1:**
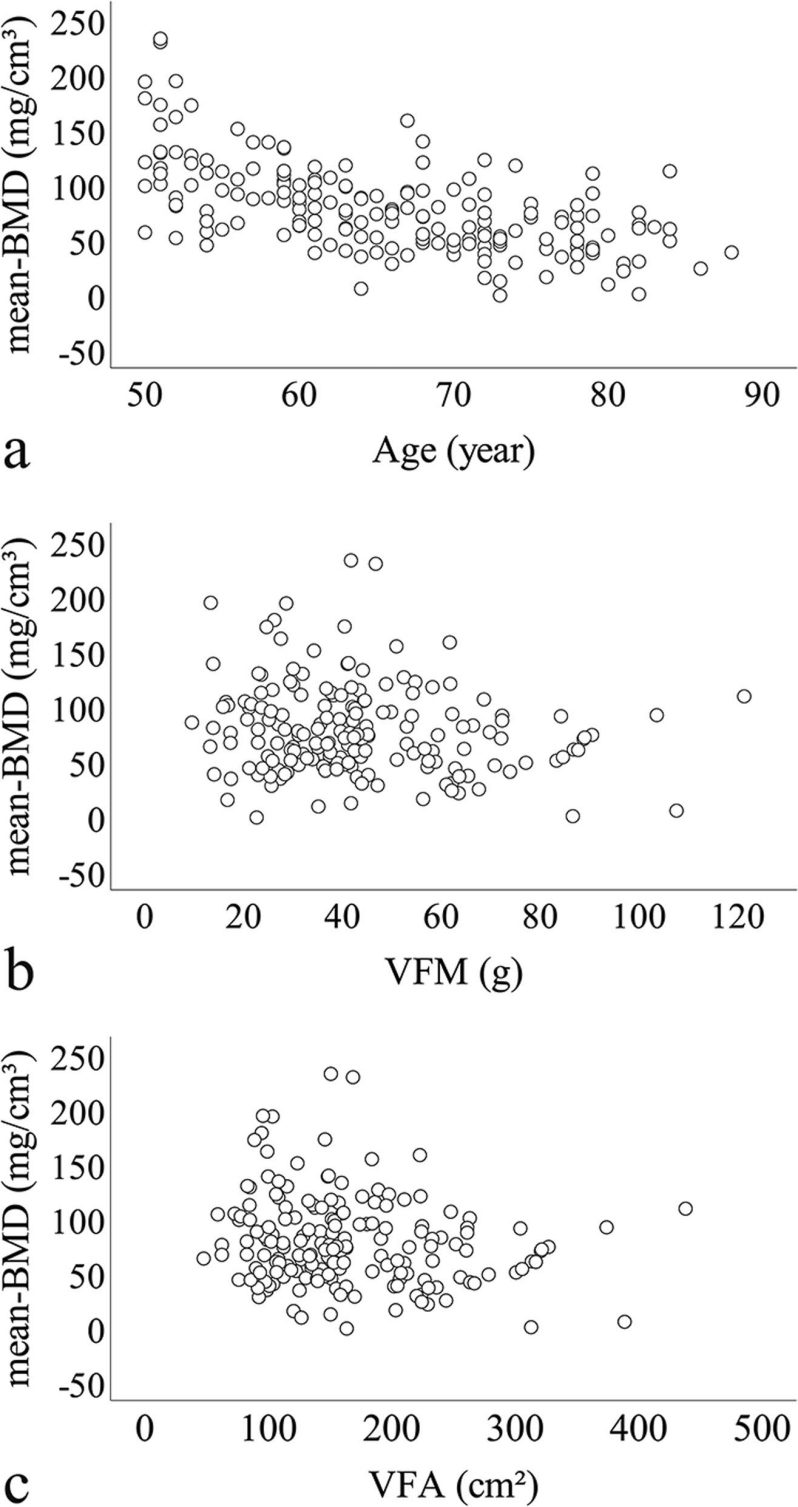
Scatter plot of correlation between age (**a**), visceral fat mass (**b**), visceral fat area (**c**) and bone mineral density

Pearson's correlation analysis indicated that BMD of lumbar spine showed a negative correlation with age (*r* = -0.590, *p* value ≤ 0.001), VFM (*r* = -0.155, *p* value = 0.040) and VFA (*r* = -0.179, *p* value = 0.017) (Fig. 1). There was no significant correlation between SFM, SFA, PMM, PMA and BMD (*p* > 0.05).

Multiple regression analysis showed that only age was significant and independent determinant of the BMD in postmenopausal women aged > 50 years with T2DM (β = -2.508, *p* ≤ 0.001) (Table 3).

**Table 3:** Univariable and multivariable linear regression analysis of influencing factors on BMD in postmenopausal women with T2DM

Variables	Univariate analysis		Multivariate analysis	
	Coefficient (95% CI)	*P*	Unstandardized Coefficient (95% CI)	*P*
Age (years)	-2.484 (-2.991, -1.976)	< 0.001	-2.508 (-3.048,1.969)	< 0.001
BMI (kg/m^2^)	-0.063 (-1.615, 1.490)	0.937		
VFM (g)	-0.314 (-0.614, -0.015)	0.040	0.212 (-0.439,0.863)	0.521
VFA (cm^2^)	-0.105 (-0.191, -0.019)	0.017	-0.048 (-0.237,0.142)	0.620
SFM (g)	-0.187 (-0.500, 0.126)	0.239		
SFA (cm^2^)	-0.043 (-0.125, 0.038)	0.297		
PMM (g)	-1.012 (-4.033, 2.008)	0.509		
PMA (cm^2^)	-0.480 (-1.511, 0.551)	0.359		

*BMI* body mass index, *VFM* Visceral fat mass, *VFA* Visceral fat area, *SFM* Subcutaneous fat mass, *SFA* Subcutaneous fat area, *PMM* Psoas major mass, *PMA* Psoas major area

## Discussion

This study was carried out to evaluate the changes of BMD and body composition in postmenopausal female patients with T2DM using QCT. This present study showed that postmenopausal patients with T2DM had higher BMI, BMD, VFM, VFA and SFM than non-diabetic postmenopausal women.

The relationship between BMI and fat mass is stronger than with LM in postmenopausal women [15]. The present results show that postmenopausal women with T2DM have higher BMI than those without diabetes, which means postmenopausal women with T2DM are more likely obese than normal elderly women. Obesity is the most common factor in the development of IR and obesity management is considered to be the primary treatment target of type 2 diabetes [16]. This result reminds us that we should pay attention to the weight management of patients with T2DM.

It is controversial about the relationship between diabetes and BMD. Some studies suggest that there is no significant difference in lumbar BMD between diabetes mellitus patients and normal glucose tolerance group in the elderly [17, 18], while some study report an increase in BMD in patients with diabetes [19]. In the present study, postmenopausal women with T2DM over 65 years old have higher BMD than those without diabetes, significant differences were observed, which is not consistent with some previous literature. Liu et al. studied 775 Han men and showed that after adjusting BMI and age, there was no significant difference in BMD of lumbar spine, femoral neck and hip among different glucose metabolism participants [17]. The reason for the inconsistent results may be that the study population, gender, and different ages patients recruited would affect BMD. In this study, only the difference in BMD between postmenopausal women with T2DM and non-diabetics was found in the age group over 65 years old. The reason for the increase of BMD in diabetic patients may be that hyperglycemia can contribute to reduced bone turnover, by reducing the number of osteoclasts, reducing their differentiation and activity, and inhibiting the resorption of bone resorption lacunae [20]. Our results also showed that BMD decreased with age in both T2DM and non-T2DM group, which was mainly due to age-related bone mass reduction, mainly related to the decrease of ovarian dysfunction and estrogen level which would lead to the imbalance of bone formation and bone resorption metabolism in postmeno pausal women [21, 22].

Compared with the non-T2DM group, the VFM and VFA of T2DM group were significantly higher. This can be explained that visceral adipose tissue (VAT) is related to IR in T2DM, which causes the release of glucose and insulin levels in the liver, leading to increase of visceral fat. Yuriko's research shows that the abdominal visceral fat of Japanese adolescents with T2DM is higher than that of the control group [23]. SN et al. also found that VAT was higher in patients with type 2 diabetes than non-diabetics [24]. Although the participants are different, it shows that visceral fat deposition in diabetic patients is more obvious than that in the non-T2DM group. Different from VAT, differences in subcutaneous adipose tissue (SAT) between the diabetic group and non-T2DM group were found only in SFM. VAT has been reported to be more strongly associated with IR than SAT [25]. In addition, obese people with normal metabolism are more likely to accumulate subcutaneous fat, and the accumulation of visceral fat is relatively less, while some people with IR, their visceral fat content is significantly increased, subcutaneous fat is less [26]. This explains why the difference in VAT is more obvious than SAT between T2DM and non-T2DM group. There are differences in SFM between T2DM and non-T2DM group, but no statistical difference in SFA, which indicates that when using QCT to evaluate subcutaneous fat in patients, both mass and area should be considered. Manoj et al. found that compared with the age-matched healthy control group, the volume of SAT and total abdominal fat in T2DM patients tended to increase [27]. On the contrary, a study from Japan showed that obese adolescents in T2DM group had lower SFA than that in simple obesity group [23]. We speculate that different results may be shown due to different study populations and different measurement parameters of subcutaneous fat. Our study also showed that the VFM and VFA differ between the two age groups, whether in T2DM group or non-T2DM group. With the growth of age, VAT gradually accumulated and increased, the results are the same as that of previous studies [28]. In elderly women, the decrease of estrogen leads to the redistribution of fat and the increase of visceral fat deposition, resulting in increased VFM and VFA [29].

Our study showed that there was no difference in the mass and area of psoas major muscle between diabetic and normal participants in postmenopausal women over 50 years old, regardless of age group. It is reported that sarcopenia, as a chronic complication of type 2 diabetes [9], is characterized by decreased skeletal muscle mass and strength, which can also affect the quality of life and increase the risk of fracture. One study [10] has shown that LM which usually represents major back muscles in women over 50 years old was significantly lower than that of women before 30 years old, and subjects in osteoporosis group had higher LM. Diabetic patients had higher BMD, but the mass and area of psoas major was similar to that of the control group. The reason why there is no statistical difference may be that our patients are postmenopausal elderly women, and the difference in muscle is not as obvious as that between the elderly patients and young people. And Ma's study suggests that it is muscle strength, not muscle mass, that is associated with osteoporosis [30], which also explains the possible reason why there is no difference in the mass and area of psoas major in this study.

Body composition is mainly composed of bone, muscle and adipose tissue. In recent years, more and more attention has been paid to the effect of body composition on BMD, but there are few studies on diabetic patients. Previous studies [31, 32] have shown that subcutaneous fat is beneficial to bone structure and bone strength, while visceral fat is the opposite in healthy people. Lumbar spine BMD is negatively associated with VAT/SAT ratio in postmenopausal T2DM, suggesting that visceral fat may be harmful to bone quality [33]. Our results showed that the age, VFA and VFM were negatively correlated with lumbar spine BMD in postmenopausal women with diabetes. However, after adjusting for BMI, only age was an independent risk factor for BMD in the study, and, no correlation was found between VAT and BMD. Previous study have shown that there is no significant correlation between VAT and BMD in the elderly over 55 years old [31]. Although the research objects are different, our results are consistent with previous studies.

### Limitations

There were some limitations in this study. The enrolled participants were relatively small, we still need to increase the sample size and enrich the age groups to provide reliable data support for the research of BMD in T2DM. Moreover, we measured the area of psoas major muscles or VAT instead of the volume, which may have an impact on the results. Additionally, the relationship between some clinical biochemical indicators such as HbA1c with BMD and body composition has not been discussed.

## Conclusions

We compared the BMD and body components distribution of the elderly women with diabetes. Our study showed that BMD is increased in elderly diabetic women. Although the VAT of diabetic patients was higher than that of normal people, there was no significant correlation between VAT and BMD. Considering that postmenopausal women with T2DM have higher visceral fat and BMI than normal postmenopausal women, we need to pay attention to their weight management. Further well-designed studies, including more clinical laboratory indicators, such as HbA1c, are warranted to evaluate the differences in body composition between patients with T2DM in different glucose metabolism status. The mechanism of increased BMD in diabetes still needs further study.

## Supplementary Information

**Additional file 1.**

## Data Availability

All data generated or analyzed during this study are included in this published article and its supplementary information files.

## Abbreviations

BMD: Bone mineral density
QCT: Quantitative CT
T2DM: Type 2 diabetes mellitus
BMI: Body mass index
VFM: Visceral fat mass
VFA: Visceral fat area
SFM: Subcutaneous fat mass
SFA: Subcutaneous fat area
PMM: Psoas major mass
PMA: Psoas major area
DXA: Dual-energy X-ray absorptiometry
IR: Insulin resistance
LM: Lean mass
HbA1c: Glycosylated hemoglobin
VAT: Visceral adipose tissue
SAT: Subcutaneous adipose tissue
