# Supplementary material for: Relationship between body composition and bone mineral density in postmenopausal women with type 2 diabetes mellitus
Source: BMC Musculoskelet Disord. 2022 Oct 3;23:893. doi: 10.1186/s12891-022-05814-8 (PMC9528089; doi:10.1186/s12891-022-05814-8)
Supplement: Supplementary file 1 — Additional file 1. [file 12891_2022_5814_MOESM1_ESM.docx]

Dear editor,

**Re:** Manuscript ID: 97ff37ed-b780-430a-a81a-bc53f557924b

**DOI:** 10.1186/s12891-022-05814-8

**Title:** Relationship between body composition and bone mineral density in postmenopausal women with type 2 diabetes mellitus

We carefully read the proofs and made some corrections, which we hope get the approval. We are sorry that we still found some mistakes in proof. Thank you for your patient reading. We have explained and replied to the modifications one by one. The modifications are as flowing:

**A major modification and** **explanation of new Table 3 and its related changes**

We added the results of the univariable linear regression analysis to Table 3, which has no any impact on the original results and we made no any changes in the results in addition to changing the standardized coefficient into the unstandardized coefficient.

*The following is an explanation of modification:*

1. The unstandardized coefficient is more consistent with the writing specification of the paper, so we have changed the standardized regression coefficient to the unstandardized coefficient, and so we changed “β=-0.595” to "β=-2.508” in the results section.

To prove the authenticity of the data, we take the screenshots of the statistical results of SPSS as follows.

2. The reason why univariable linear regression analysis results was not added before was that the results were consistent with the correlation analysis result. However, we have thought over and over again and finally decided to add this result after careful consideration. Although the reviewer did not raise this issue, we believe that such modification makes our results more rigorous, convincing, and more able to maintain the scientific integrity of this paper than before. Thus, the term “univariable” is added to the statistical method. A new Table 3 has been uploaded to the attachment of proofreading system in word format.

Some changes related to new Table 3 are as follows:

**Abstract**

**Methods**

“Multiple linear regression analysis was” has been changed to “Univariable and multivariable linear regression analysis were”.

**Methods**

**Statistical analysis**

“A multivariable linear regression model was developed to identify the factors contributing to BMD”

has been changed to “Univariable and multivariable linear regression model were developed to identify the factors contributing to BMD”.

“In multiple linear regression, the average BMD of lumbar 1–3 vertebrae……”

has been changed to “In linear regression, the average BMD of lumbar 1–3 vertebrae ……”

**Other modification and explanation (In the order of AQ and section headings)**

**AQ1** Please check the author corresponding email address if captured correctly.

**Reply:** The email addresses of corresponding authors have been checked.

The addresses of the authors have been changed to a unified format.

**AQ2** As per our journal style, article titles should not include capitalised letters unless these are proper nouns/acronyms. We have therefore used the article title “Relationship between body composition and bone mineral density in postmenopausal women with type 2 diabetes mellitus” as opposed to “Relationship between Body Composition and Bone Mineral Density in Postmenopausal Women with Type 2 Diabetes Mellitus” as given in the submission system. Please check if this is correct.

**Reply:** We have checked that there is no error in the title that you have modified.

**Keywords**

Please help me change “Type 2 diabetes” to "Type 2 diabetes mellitus", which is more standardized and scientific.

**Abbreviations**

PMA Psoas major area;

“;” Should be deleted.

**AQ3** Please check the affiliations if captured correctly.

**Reply:** Please help me delete Aff4, because Aff4 and Aff3 are duplicate. I'm sorry I didn't find the button to delete Aff4.

**AQ4** Please check if the section headings are assigned to appropriate levels.

**Reply:** We have checked that the section headings has been assigned to the appropriate levels. Thank you for your reminding.

**Background**

“Aging usually leads to loss of lean mass” has been changed to

“Aging usually leads to loss of lean mass (LM)”

**Methods**

**Patients**

“patients with FPG” should be changed to " patients with fasting plasma glucose (FPG)"

"HbA1c ≥ 6.5%" should be changed to "glycosylated hemoglobin (HbA1c) ≥ 6.5%"

“The treatment, levels of glycosylated hemoglobin (HbA1c)” should been changed to “The treatment, levels of HbA1c”

**Body compositions measurement**

Measurements of ……at the middle level of navel on the image with a reconstruction thickness of 1 mm. " “at the middle level of navel “should be deleted because similar content has already been mentioned in the previous paragraph.

**Results**

**Baseline characteristics of participants**

136 postmenopausal females without diabetes aged 5091

“5091” should be changed to “50-91”

**Table2**

*P_4_* “65y in T2DM group vs 65y in non-T2DM group”

should be changed to
“> 65y in T2DM group vs > 65y in non-T2DM group”

**Correlation between age, BMI, and body component variables and BMD in T2DM group**

It is more appropriate to change "and" in the section subtitle to ",".

Last sentence-There was no significant difference between SFM, SFA, PMM, PMA and BMD (p > 0.05)

“difference” should be changed to “correlation”. I'm sorry for the mistake about this word.

**AQ5** Figure 1 contains text below the minimum required font size of 6pts inside the artwork, and there is no sufficient space available for the text to be enlarged. Please provide replacement figure file.

**Reply:** Thank you for reminding me that the picture is really not clear enough, and the replacement figure has been uploaded to the attachment in the proof system.

**Discussion**

**The first paragraph-first sentence-** This study was carried out to evaluate the changes of BMD and body composition in postmenopausal female patientss with T2DM using QCT.

“patientss” should be changed to “patients”. We are sorry about the spelling mistake of this word.

**The second paragraph-first sentence-** “The relationship between BMI and fat mass is stronger than with lean mass (LM)”

should be changed to “The relationship between BMI and fat mass is stronger than with LM”

It is controversial about the relationship between diabetes and BMD.

Please help me add a space before "It" or make a new paragraph start from this sentence.

**A sentence after Reference 17-**

The reason for the inconsistent results may be that the study population, gender, and different agess

“agess” should be changed to “ages”. I'm sorry about the spelling mistakes.

**Authors’ contributions**

Please help me add “:” after "Shao-qiang He".

**AQ6** Citation details for reference [9] is incomplete. Please supply the "Page range" of this reference. Otherwise, kindly advise us on how to proceed.

**Reply:** I have added the page number of the reference whose doi was 10.1186/s13098-018-0326-5.

**AQ7** Please provide the complete bibliographic details of this reference.

**Reply:** We have improved the format of references whose doi was 10.2337/db17-1871-2152.

Zhang Z-Y. Lumbar BMD is negatively associated with VAT/SAT ratio in postmenopausal T2DM Evaluated by QCT. Diabetes 2017;66 Suppl1: A561.
